# Mobilization studies in mice deficient in sphingosine kinase 2 support a crucial role of the plasma level of sphingosine-1-phosphate in the egress of hematopoietic stem progenitor cells

**DOI:** 10.18632/oncotarget.19514

**Published:** 2017-07-24

**Authors:** Mateusz Adamiak, Lakshman Chelvarajan, Kevin R. Lynch, Webster L. Santos, Ahmed Abdel-Latif, Mariusz Z. Ratajczak

**Affiliations:** ^1^ Stem Cell Institute at James Graham Brown Cancer Center, University of Louisville, Louisville, KY, USA; ^2^ Department of Regenerative Medicine, Warsaw Medical University, Warsaw, Poland; ^3^ Division of Cardiovascular Medicine, Gill Heart Institute, University of Kentucky, Lexington, KY, USA; ^4^ Department of Pharmacology University of Virginia, Charlottesville, VA, USA; ^5^ Department of Chemistry, Center for Drug Discovery, Virginia Tech, Blacksburg, VA, USA

**Keywords:** S1P, Sphk1, Sphk2, stem cell mobilization

## Abstract

Sphingosine-1-phosphate (S1P) is a bioactive lipid involved in cell signaling and, if released from cells, also plays a crucial role in regulating the trafficking of lympho-hematopoietic cells, including primitive hematopoietic stem/progenitor cells (HSPCs). It has been demonstrated that S1P chemoattracts HSPCs, and its level in peripheral blood creates a gradient directing egress of these cells during mobilization. In this paper we analyzed hematopoiesis in mice deficient in sphingosine kinase 2 (Sphk2-KO mice) and studied the effect of this mutation on plasma S1P levels. We found that Sphk2-KO mice have normal hematopoiesis, and, in contrast to Sphk1-KO mice, the circulating S1P level is highly elevated in these animals and correlates with the fact that HSPCs in Sphk2-KO animals, also in contrast to Sphk1-KO animals, show enhanced mobilization. These results were recapitulated in wild type (WT) animals employing an Sphk2 inhibitor. We also administered an inhibitor of the S1P-degrading enzyme S1P lyase, known as tetrahydroxybutylimidazole (THI), to WT mice and observed that this resulted in an increase in S1P level in PB and enhanced mobilization of HSPCs. In sum, our results support a crucial role for S1P gradients in blood plasma in the mobilization process and indicate that small-molecule inhibitors of Sphk2 and Sgpl1 could be employed as mobilization-facilitating compounds. At the same time, further studies are needed to explain the unexpected effect of Sphk2 inhibition on increasing S1P levels in plasma.

## INTRODUCTION

Evidence has accumulated that the bioactive lipid sphingosine-1-phosphate (S1P) is an important chemoattractant for lymphocytes [1, 2] and, together with ceramide-1-phosphate (C1P), plays a pivotal role in the trafficking of hematopoietic stem/progenitor cells (HSPCs) [2, 3]. S1P has been reported to play a role both in mobilization of HSPCs from bone marrow (BM) into peripheral blood (PB) [4–6] and in the reverse phenomenon of homing to BM of cells circulating in PB [7]. S1P is highly expressed in tissue fluids, such as blood and lymph, and in PB its most important sources are red blood cells, albumin, and apoM bound to high-density lipoproteins (HDL) [8]. The high concentration of S1P in PB creates an important chemotactic gradient across the endothelium between BM and PB [2].

As a bioactive lipid and a phosphorylated product of sphingosine, S1P is an important intracellular second messenger and, if released from the cells, is a ligand for five different G protein-coupled S1P receptors (S1P1-5) [2, 9]. Of these receptors, S1P receptor type 1 (S1P1) and (as recently demonstrated) S1P3 [2, 10–12] play an important role in the trafficking of lympho/hematopoietic cells. An important source of sphingosine is ceramide, and sphingosine is derived from ceramides in a ceramidase-dependent manner. The process of sphingosine phosphorylation to produce S1P is governed by sphingosine kinases. Two forms of sphingosine kinase —type 1 (Sphk1) and type 2 (Sphk2)—have been described [2, 13, 14]. Sphk1 is found in the cytosol of eukaryotic cells and shifts to the plasma membrane upon activation, while Sphk2 is localized to the cell nucleus [15]. As previously demonstrated, mice with Sphk1 knockout are poor HSPC mobilizers [5] and have impaired homing of HSPCs after transplantation [16].

On the one hand, S1P can be inactivated by dephosphorylation with sphingosine phosphate lyase (Sgpl1) [17]. Inhibition of Sgpl1 with tetrahydroxybutylimidazole (THI), which is a small-molecule inhibitor of this enzyme, results in an increase in S1P levels in PB and leads to release of c-kit^+^ stem cells from BM in a mouse model of acute myocardium infarction [18]. On the other hand, Sgpl1 inhibition by THI has been related to its immunosuppressive role as a result of retention of lymphocytes in lymphoid organs, which results in lymphopenia [19].

Recently published results suggest a crucial role of the S1P level in PB in directing egress of HSPCs from BM into PB [20, 21]. To better address this question we performed mobilization studies in Sphk2-KO mice and WT mice exposed to inhibitors of Sphk2 and Sgpl1 to modulate the level of S1P in PB.

We show what is counterintuitive, that is, the S1P level in Sphk2-KO mice and wild type (WT) mice exposed to a small-molecule Sphk2 inhibitor is elevated relative to control animals and Sphk1-KO animals. The S1P plasma level was also increased in THI-treated animals. Moreover, our mobilization experiments lend further support to there being a crucial role of the S1P blood level in egress of HSPCs from BM into PB, as enhanced mobilization in Sphk2-deficient mice and WT mice exposed to Sphk2 and Sgpl1 inhibitors was correlated with an increased S1P plasma level but not with significant changes in the level of CXCL12 (stromal-derived factor 1; SDF-1).

## RESULTS

### Sphingosine kinase 2-deficient (Sphk2-KO) mice show enhanced mobilization

**Figure 1:**
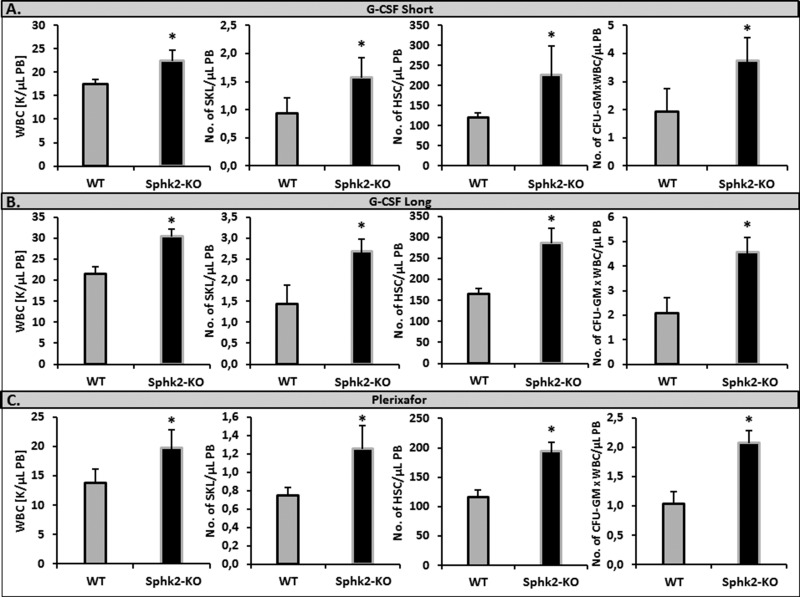
Sphk2-KO mice show enhanced mobilization with G-CSF and Plerixafor Mononuclear cells were isolated from WT and SphK2-KO mice after 3 days (Panel **A**) or 6 days (Panel **B**) of G-CSF mobilization (100 μg/kg per day, subcutaneously) or after Plerixafor-induced mobilization (5 mg/kg, intraperitoneal injection, Panel **C**). The numbers of WBCs, SKL (Sca-1^+^ c-kit^+^ Lin^−^) cells, HSCs (Sca-1^+^ CD45^+^ Lin^−^), and CFU-GM clonogenic progenitors were evaluated from PB. Results from two separate experiments are pooled together. *P ≤ 0.05.

It has been reported that the mobilization of HSPCs is impaired in sphingosine kinase 1-deficient (Sphk1-KO) mice, due to a low plasma level of S1P [5, 22]. Since Sphk2 is another enzyme involved in S1P synthesis, we became interested in whether a similar effect occurs in Sphk2-KO animals. However, to our surprise, as shown in Figure 1, we found that Sphk2-KO show enhanced mobilization in response to a short (3-day) G-CSF-, a long (6-day) G-CSF-, or Plerixafor-induced mobilization.

Supplementary Figure 1 shows that the Sphk2-KO mice employed in our studies have normal PB cell counts (Supplementary Figure 1A), red blood cell parameters (Supplementary Figure 1B), numbers of bone marrow-residing HSPCs (Supplementary Figure 1C), and clonogenic progenitors (Supplementary Figure 1D) under steady-state conditions compared with WT animals.

### Changes in S1P and CXCL12 level in Sphk1-KO and Sphk2-KO mice

**Figure 2:**
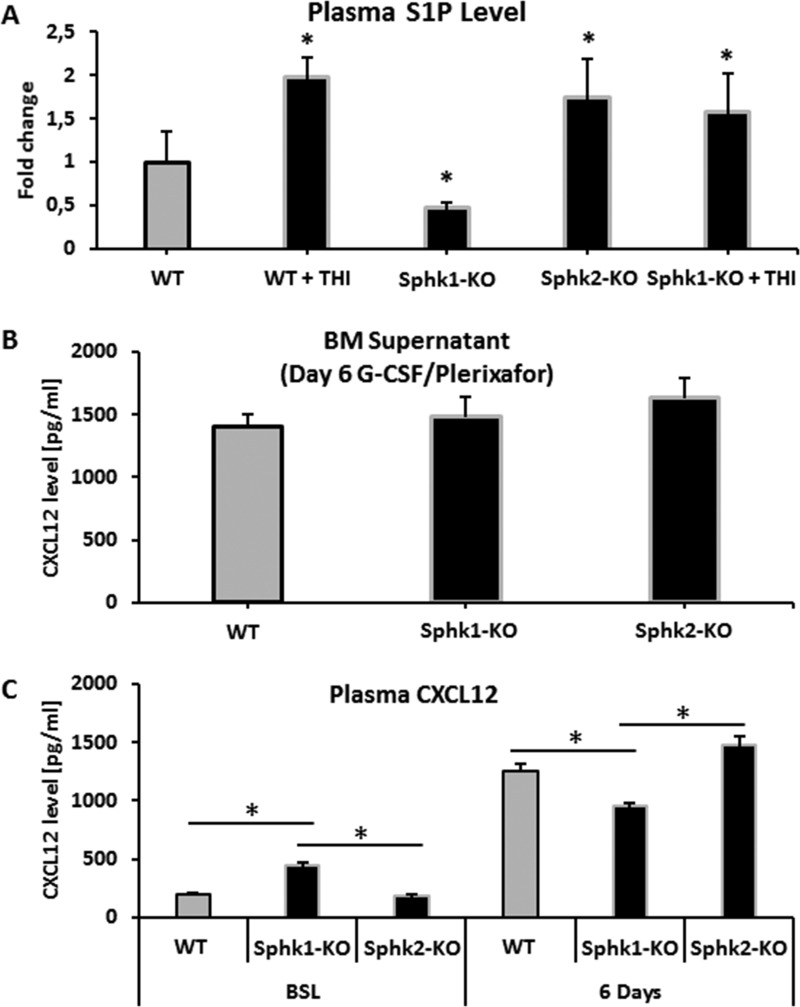
Measurements of S1P and CXCL12 levels Panel (**A**) Steady-state S1P plasma level in WT, Sphk1-KO, Sphk2-KO, and Sphk1-KO mice exposed to THI. Panel (**B**) CXCL12 concentration in conditioned media from BM after 6 days of G-CSF mobilization in Sphk1-KO and Sphk2-KO animals. Panel (**C**) Left: the plasma level of CXCL12 in Sphk1-KO and Sphk2-KO animals under steady-state conditions. Right: the plasma level of CXCL12 in Sphk1-KO and Sphk2-KO animals mobilized for 6 days with G-CSF. Results from two separate experiments are pooled together.

Since the mobilization of HSPCs from BM into PB correlates with the plasma level of S1P, we measured the S1P level in WT, Sphk1-KO and Sphk2-KO animals. Figure 2 shows that, in contrast to Sphk1-KO mice, the plasma level of S1P is significantly elevated in Sphk2-KO mice. Thus, the increase in S1P in the PB of Sphk2-mutant animals suggests the mobilization-promoting gradient for HSPCs. However, a molecular explanation of the increase in S1P is not clear at this time and requires further study. The S1P plasma level was also increased as expected in THI-treated Sphk1-KO mice. Interestingly Sphk2-KO mice, in contrast to Sphk1-KO and WT animals, also had elevated levels of C1P in PB (Supplementary Figure 2), which indicates that changes in S1P level in PB in Sphk2-KO mice are echoed by changes in the level of another chemotactic factor for HSPCs, C1P [2, 23].

We also evaluated CXCL12 levels in conditioned media harvested from BM supernatants (Figure 2B) and PB plasma (Figure 2C) harvested from WT, Sphk1-KO, and Sphk2-KO mice mobilized for 6 days with G-CSF (Figure 2B). While there were no major changes in CXCL12 levels in the conditioned media and plasma of G-CSF-mobilized animals, the baseline CXCL12 level was enhanced in the plasma of Sphk1-KO mice. Of note, this increase in CXCL12 level in PB plasma reached 1.5 ng/ml, which is still a very low concentration that, as we previously demonstrated, is not alone sufficient to promote migration of HSPCs [7, 20].

### Inhibition of S1P lyase (Sgpl1) by tetrahydroxybutylimidazole (THI) enhances the S1P level in PB and enhances mobilization in WT animals

**Figure 3:**
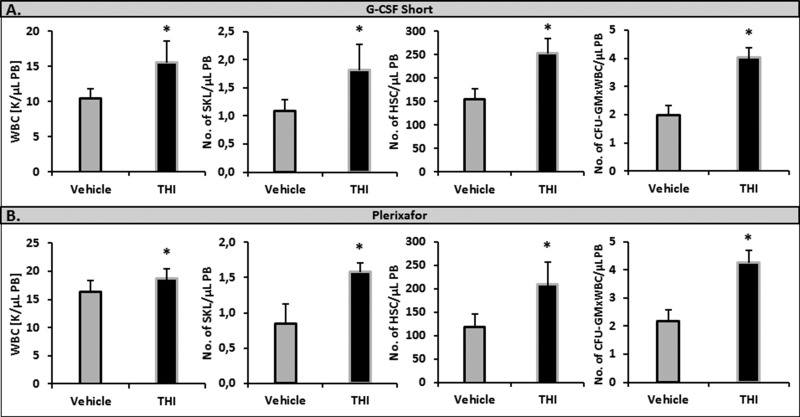
Impact of an S1P lyase (Sgpl1) inhibitor on the mobilization of HSPCs WT mice received mobilizing agents and tetrahydroxybutylimidazole (THI) administered ad libitum in water or vehicle. Mononuclear cells were isolated after 3 days of G-CSF- (Panel **A**) or Plerixafor-induced mobilization (Panel **B**). The numbers of WBCs, SKL (Sca-1^+^ c-kit^+^ Lin^−^) cells, HSCs (Sca-1^+^ CD45^+^ Lin^−^), and CFU-GM clonogenic progenitors were evaluated in PB. Results from two separate experiments are pooled together. *P ≤ 0.05.

Since Sgpl1 degrades S1P, we became interested in whether inhibition of this enzyme would increase mobilization of HSPCs in WT animals. We found that inhibition of Sgpl1 did increase the S1P level in PB in both WT [19, 20, 25] and Sphk1-KO animals (Figure 2A). This increase in plasma S1P level was correlated with enhanced mobilization in WT mice exposed to the Sgpl1 inhibitor THI. To support this Figure 3 shows enhanced mobilization of HSPCs in WT animals pretreated with THI in response to G-CSF and Plerixafor administration.

### Inhibition of Sphk2 by small-molecule inhibitor of SLM6041434 enhances the S1P level in PB and enhances mobilization in WT animals

**Figure 4:**
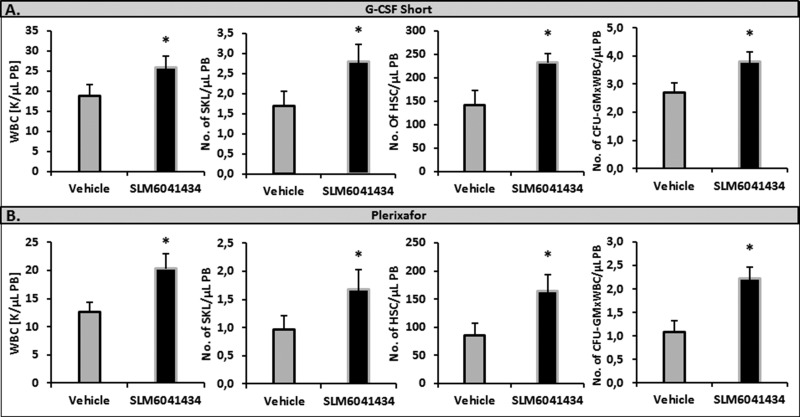
Impact of an Sphk2 inhibitor on the mobilization of HSPCs WT mice received mobilizing agents and SLM6041434 compound (or instead of SLM6041434 compound, control mice received vehicle). Mononuclear cells were isolated after 3 days of G-CSF- (Panel **A**) or Plerixafor-induced mobilization (Panel **B**). The numbers of WBCs, Sca-1^+^ c-kit^+^ Lin^−^ cells (SKL cells), HSCs (Sca-1^+^ CD45^+^ Lin^−^), and CFU-GM clonogenic progenitors were evaluated in PB. Results from two separate experiments are pooled together. *P ≤ 0.05.

Moreover, corroborating our results shown in Figure 2A, it has been recently reported that the S1P level is elevated in blood of WT mice exposed to a small-molecule inhibitor of Sphk2, SLM6041434 [24]. Therefore, to investigate whether WT mice exposed to SLM6041434 better mobilize HSPCs, we performed mobilization studies in these animals. Figure 4 shows that WT mice exposed to SLM6041434 better mobilized HSPCs in response to G-CSF and Plerixafor than control animals not exposed to this Sphk2 inhibitor.

### In comparison with WT animals, Sphk2-KO mice show defective BM engraftment of HSPCs

In our previous work we demonstrated that S1P enhances CXCL12-mediated homing and even compensates in case of any deficiency in homing to BM of HSPCs infused into PB [26]. Based on our current observation that the S1P plasma level is high in Sphk2-KO animals, we asked whether this increase prevents homing to BM of HSPCs. To address this question we performed WT HSPC transplants in WT and Sphk2-KO animals.

**Figure 5:**
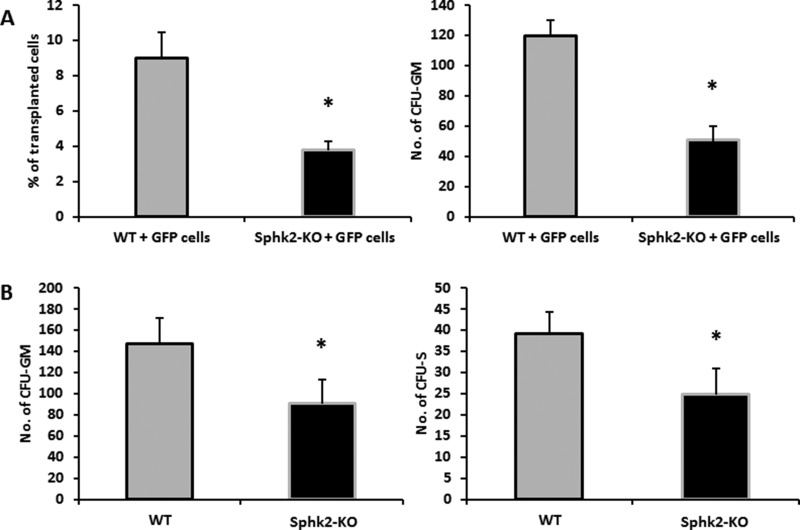
Defects in homing and short-term engraftment of HSPCs in Sphk2-KO mice Panel (**A**) Lethally irradiated mice (six mice per group) were transplanted with 7 × 10^6^ bone marrow mononuclear cells (BMMNCs) from B6-GFP^+^ mice. Twenty-four hours after transplantation, femoral BMMNCs were harvested, the number of GFP^+^ cells was evaluated by FACS (Panel A, left), and the clonogenic CFU-GM progenitors were enumerated in an *in vitro* colony assay (Panel A, right). No colonies were formed in lethally irradiated and non-transplanted mice (irradiation control). Panel (**B**) Lethally irradiated mice (six per group) were transplanted with 2.5 × 10^5^ BMMNCs from WT mice. Twelve days after transplantation, femoral BMMNCs were harvested to evaluate the number of CFU-GM colonies in clonogeneic assays (Panel B, left), and the spleens were removed to enumerate the number of CFU-S colonies (Panel B, right). The data represent the combined results from two independent experiments for each panel. *p < 0.005.

**Figure 6:**
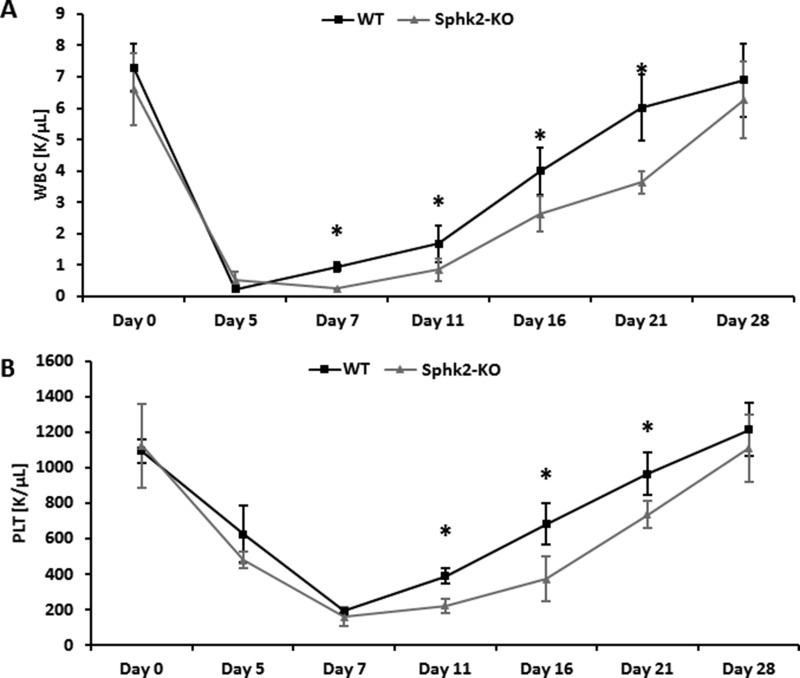
Defects in engraftment of HSPCs in Sphk2-KO mice Lethally irradiated WT and Sphk2-KO mice were transplanted with 2 × 10^5^ BMMNCs from WT cells. White blood cells (Panel **A**) and platelets (Panel **B**) were counted before irradiation and at intervals 5, 7, 11, 16, 21, and 28 days after irradiation. The data represent the combined results from two independent experiments for each panel. *p < 0.005.

We found that Sphk2-KO mice transplanted with BMMNCs labeled with green immunofluorescence protein (GFP) showed impaired homing of GFP^+^ BM cells compared with WT mice 24 hours later (Figure 5A). Moreover, 12 days after transplantation Sphk2-KO animals transplanted with GFP^+^ BMMNCs had lower numbers of clonogenic CFU-GM colonies in BM and lower numbers of CFU-S colonies than control WT animals (Figure 5B). Finally, Sphk2-KO animals transplanted with WT BMMNCs showed delayed recovery of neutrophil and platelet counts compared with control mice (Figure 6).

## DISCUSSION

The seminal observation of this report is that Sphk2-null mice and mice exposed to Sphk2 and Sgpl1 inhibitors have elevated S1P levels in PB and show enhanced mobilization of HSPCs. Thus, our results support the conclusion that S1P is a major PB chemoattractant for HSPCs [2, 4, 5, 16, 20]. We also demonstrate that in the reverse process of homing of HSPCs from PB into BM, an elevated S1P level in PB, as seen in Sphk2-KO animals, may impair hematopoietic cell homing and engraftment. Overall, our results provide further support for the presence of a “tug of war” between BM and PB mediated by an S1P gradient that affects trafficking of HSPCs [27].

S1P has been proposed to play a role in trafficking of lymphocytes [1, 2], and more recently this trafficking has been found to include egress of hematopoietic progenitor cells form BM into PB [4–6]. Specifically, our group and others have demonstrated the pivotal role of S1P in egress of both hematopoietic stem cells and hematopoietic progenitors from BM into PB in the process of pharmacological mobilization induced by G-CSF or Plerixafor [5, 20]. We also proposed the concept that the S1P level is already very high in circulating PB under steady-state conditions, which supports the notion that HSPCs are actively retained in the BM microenvironment by the CXCL12–CXCR4 and VCAM-1–VLA4 axes to prevent their egress into PB in response to an S1P gradient [21, 28]. The S1P level in PB may also increase during mobilization in response to the release of this chemoattractant from red blood cells and platelets in response to activation of the complement and coagulation cascades [2, 20, 21]. Both of these cascades are crucial in induction of mobilization processes in response to pharmacology, inflammation, stress, or tissue injury [6, 29–33].

Interestingly an increase in S1P level alone in response to red blood cell hemolysis (for example, induced by administration of phenylhydrazine (PHZ), when neither complement nor coagulation cascades are activated) does not significantly induce egress of HSPCs into the circulation [21]. As reported, the doubling of S1P level in PB from damaged erythrocytes in response to PHZ-induced hemolysis only marginally increased the number of HSPCs circulating in PB. However, if mice are exposed to PHZ together with the CXCR4 blocking agent, Plerixafor, a robust synergistic increase in the number of mobilized HSPCs occurred [21]. This finding supports the concept that HSPCs first have to be released from BM niches by blocking the CXCL12–CXCR4 retention signal in order to respond to the S1P gradient between BM and PB. This is why induction of a proteolytic [34] and lipolytic [35] microenvironment in BM initiated by activation of the complement cascade[7, 8] is crucial to attenuating the biological function of the retention axes for HSPCs in BM and facilitates their egress into PB in response to an S1P gradient [7, 8, 34, 35].

S1P is synthesized by phosphorylation of sphingosine by the kinases Sphk1 and Sphk2 and is degraded by Sgpl1 or S1P phosphatase or is extruded by S1P transporters, such as sphingolipid transporter 2 (Spns2) [2, 13, 14, 17, 36]. Mice that are deficient in both Sphk1 and Sphk2 die in utero with no detectable S1P, which suggests an important role for S1P in development [9]. However, single-knockout mice are viable. Interestingly, while Sphk1-KO mice display a decrease by ∼50% of the S1P level circulating in PB [37], mice with Sphk2 deficiency have a somewhat unexpected 2–4-fold increase in S1P level in PB plasma and whole blood [24, 38]. It has already been reported that Sphk1-KO mice are poor mobilizers of HSPCs [5], which nicely correlates with the low level of blood plasma S1P in these animals [37]. Based on the fact that Sphk2-KO mice have elevated levels of S1P in PB, we performed mobilization studies in these animals and demonstrated that, as expected, they show enhanced mobilization after 3-day or 6-day administration of G-CSF and after mobilization by employing the CXCR4 receptor antagonist Plerixafor.

This increase in S1P level in peripheral blood in Sphk2-KO mice correlating with their enhanced mobilization state was reproduced using a nontoxic dose of the Sphk2 inhibitor SLM6041434. In our hands, WT mice exposed to this compound better mobilized HSPCs after either G-CSF or Plerixafor administration, and we confirmed that this correlated with an increase in S1P level in PB. We obtained additional support for the pivotal role of an increase in S1P level in circulating blood in the egress of HSPCs from BM by employing the commercially available Sgpl1 inhibitor tetrahydroxybutylimidazole (THI) [18, 19, 25]. WT mice exposed to this compound again turned out to better mobilize HSPCs than control animals not exposed to THI. This observation supports our recent results performed in mice after experimental acute myocardium infarction (AMI) in which administration of THI led to an increase in S1P level in PB and mobilization of c-kit^+^ stem cells from BM [18]. This enhanced mobilization of stem cells resulted in an increase in angiogenesis, improved recovery of cardiac functional parameters, and reduction in scar size [18].

The increased baseline level of S1P in Sphk2-KO mice is intriguing. As reported recently, mass-labeled S1P is cleared more slowly in these mutant animals, which suggests that Sphk2 deficiency results also in decreased clearance of circulating S1P and strongly suggests that this enzyme may have additional yet-unrecognized functions independent of the intracellular synthesis of S1P [24]. Several explanations for this phenomenon have been proposed [24], for example, a compensatory increase in Sphk1 activity or a so-far-uncharacterized S1P-degrading capacity as a lyase or phosphatase. However none of these explanations is conclusive, and this phenomenon requires further study.

A striking aspect of S1P biology is its compartmentalization between tissues and circulating fluids [24, 39, 40]. Specifically, S1P levels are, as mentioned above, significantly higher in PB and lymph than in tissues, for example, in the BM microenvironment [8]. However, as we previously demonstrated, the S1P level is upregulated in BM after conditioning for hematopoietic transplant by radio- or chemotherapy [7]. This effect supports the presence of a dynamic tug of war between the S1P gradient (between PB and BM) and the retention axes. The important role of S1P in homing of HSPCs, which functionally supports the CXCL12–CXCR4 homing axis, has been demonstrated after transplantation of CXCR4-deficient HSPCs into Sphk1-KO animals [16]. In the current paper we support this tug-of-war concept between the S1P gradient between BM and PB and the retention axes and demonstrate that a high level of S1P in PB, as seen in Sphk2-KO mice, impairs both homing and short-term engraftment of HSPCs in lethally irradiated WT mice.

Since a role for S1P has been proposed in the trafficking of malignant hematopoietic cells, targeting the S1P axis may also become an important part of anti-leukemic therapy [2]. The mechanism of Sphk2 inhibitors in leukemia is mainly based on increasing autophagic death, as seen for example in T cell-acute lymphocytic leukemia [41, 42], or a decrease in cell proliferation and induction of apoptosis in multiple myeloma cells [43]. Our findings reported in this work show that there are most likely clear differences in the Sphk2 requirement between normal and malignant hematopoietic cells, as Sphk2 deficiency does not affect normal hematopoiesis in Sphk2-KO mice, and we did not observe any negative effects of an Sphk2 inhibitor on clonogenic proliferation of normal HSPCs in the *in vitro* toxicity studies performed.

In conclusion, our novel results support the overall concept that the S1P level in PB is a major chemoattractant for BM-residing HSPCs and the presence of a tug of war between BM and PB due, in part, to this bioactive sphingolipid. Thus, changes in S1P gradient direct mobilization of HSPCs and may affect BM homing of these cells after transplantation. Moreover, drugs that inhibit Sphk2 activity (e.g., SLM6041434) or inhibit Sgpl1 (e.g., THI) could increase our armamentarium of available HSPC-mobilizing drugs. Since it has also been demonstrated that another bioactive sphingolipid, C1P, also directs trafficking of HSPCs, and its level is elevated in SphK2-KO animals, further studies are needed to study the relationship between the synthesis of both phosphosphingolipids.

## MATERIALS AND METHODS

### Animals

In our experiments we employed pathogen-free, 6- to 8-week-old C57BL/6J (WT), B6N.129S6-*Sphk1tm1Rlp*/J (Sphk1-KO) and B6N.129S6-*Sphk2tm1Rlp*/J (Sphk2-KO) female mice established by Rick Proia [13, 16] and purchased from the Jackson Laboratory (Bar Harbor, ME, USA) at least 2 weeks before the experiments. Animal studies were approved by the Animal Care and Use Committee of the University of Louisville (Louisville, KY, USA).

### Drug administration

For Sphk2 inhibition, mice were injected i.p. with 5 mg/kg SLM6031434 or an equal volume of vehicle (2% solution of hydroxypropyl-*β*-cyclodextrin; Cargill Cavitron 82004; Cargill Inc., Cedar Rapids, IA). To inhibit S1P lyase, mice received vehicle or 25 mg/L tetrahydroxybutylimidazole THI (Sigma-Aldrich, St. Louis, MO) administered ad libitum in water containing 5% dextrose (to improve palatability). THI is a Food and Drug Administration-approved caramel food coloring additive shown to inhibit S1P lyase when administered orally to mice [19, 25].

### Murine bone marrow-derived mononuclear cells

Bone marrow-derived mononuclear cells (BMMNCs) were obtained by flushing femurs and tibias of pathogen-free experimental mice. Cells were lysed with BD Pharm Lyse buffer (BD Biosciences, San Jose, CA, USA) to remove red blood cells, washed, and resuspended in appropriate media for further analysis [35, 44].

### Mobilization

Experimental mice were injected subcutaneously (s.c.) with 100 μg/kg G-CSF (Amgen, Thousand Oaks, CA, USA) daily for 3 (short mobilization) or 6 (long mobilization) days and one dose at 5 mg/kg (i.p.) of Plerixafor (AMD3100) (Sigma-Aldrich). At 6 h after the last G-CSF administration or at 1 h after Plerixafor injection mice were bled from the retro-orbital plexus for hematology analysis, and PB was obtained from the vena cava with a 25-gauge needle and 1-ml syringe containing 50 μl of 100 mM ethylenediaminetetraacetic acid (EDTA; Quality Biological Inc., Gaithersburg, MD, USA). Mononuclear cells (MNCs) were obtained by hypotonic lysis of RBCs in BD Pharm Lyse buffer [35, 44].

### Fluorescence-activated cell sorting (FACS) analysis

The following monoclonal antibodies were used to perform staining of Lin^−^/Sca-1^+^/c-Kit^+^ (SKL) cells and Lin^−^/Sca-1^+^/CD45^+^ hematopoietic stem cells (HSCs): FITC–anti-CD117 (also known as c-Kit, clone 2B8; BioLegend, San Diego, CA, USA) and PE–Cy5–anti-mouse Ly-6 A/E (also known as Sca-1, clone D7; eBioscience, San Diego, CA, USA). All anti-mouse lineage marker [5] antibodies, including anti-CD45R/B220 (clone RA3-6B2), anti-Ter-119 (clone TER-119), anti-CD11b (clone M1/70), anti-T cell receptor β (clone H57-597), anti-Gr-1 (clone RB6-8C5), anti-TCRγδ (clone GL3), and anti-CD45 (clone 30-F11), were purchased from BD Biosciences and conjugated with PE as described [35, 44]. Staining was performed in RPMI 1640 medium containing 2% FBS. All monoclonal antibodies (mAbs) were added at saturating concentrations, and the cells were incubated for 30 min on ice, washed twice, and analyzed with an LSR II flow cytometer (BD Biosciences).

### Evaluation of HSPC mobilization

For evaluation of circulating colony-forming unit-granulocyte/macrophage (CFU-GM) and SKL cells, the following formulas were used: (number of white blood cells [WBCs]) x number of CFU-GM colonies)/number of WBCs plated = number of CFU-GM per μl of PB; and (number of WBCs x number of SKL cells)/number of gated WBCs = number of SKL cells per μl of PB [35, 44].

### PB parameter counts

To obtain leukocyte and RBC counts, 50 μl of PB was taken from the retro-orbital plexus of the mice and collected in microvette EDTA-coated tubes (Sarstedt Inc., Newton, NC, USA), and samples were analyzed within 2 h of collection on a HemaVet 950 analyzer (Drew Scientific Inc., Waterbury, CT, USA) [35, 44, 45].

### Clonogenic *in vitro* assay

RBCs from PB or BM were lysed with BD Pharm Lyse buffer. Nucleated cells were subsequently washed, counted, and resuspended in human methylcellulose base medium provided by the manufacturer (R&D Systems, Minneapolis, MN, USA). To evaluate the number of clonogenic progenitor cells, BMMNCs were supplemented with erythropoietin (5 U/ml; Stemcell Technologies, Vancouver, BC, Canada) plus stem cell factor (SCF; 5 ng/ml; R&D Systems) and resuspended in methylcellulose base medium (for determining the number of burst-forming units-erythroid; BFU-E)(R&D Systems). For determining the number of megakaryocytic progenitors (CFU-Meg) BM-MNC were supplemented with thrombopoietin (100 ng/ml; Gibco Thermo Fisher Scientific, Waltham, MA, USA) plus mIL-3 (10 ng/ml; ProSpec-Tany Technogene Ltd., East Brunswick, NJ, USA), and resuspended in plasma clots. For determining number of granulocyte/monocytic colonies (CFU-GM) BM-MNC were supplemented with 25 ng/ml recombinant murine granulocyte macrophage colony-stimulating factor (mGM-CSF; Millipore, Billerica, MA, USA) and 10 ng/ml recombinant murine interleukin 3 (mIL-3; Millipore) and cultured in in methylcellulose base medium. Cultures were incubated for 7 to 14 days (37°C, 95% humidity, and 5% CO_2_), at which time they were counted under an inverted microscope (Olympus CK40; Olympus, Shinjuku, Tokyo, Japan) [16, 44, 45].

### Quantitation of S1P and C1P levels

PB samples were obtained from the retro-orbital plexus of the mice into tubes containing 1:5 ratio of EDTA:CTAD. Plasma was isolated by centrifuging whole blood for 10 min at 700 *g*. Supernatant was then removed and centrifuged at 10,000 *g* for 10 min to remove platelets, and the supernatant was then used for lipid measurements. Lipids were extracted from plasma, supernatant using acidified organic solvents, as previously described [46, 47]. An analysis of S1P and C1P was carried out using a Shimadzu UFLC coupled with an AB Sciex 4000-Qtrap hybrid linear ion trap triple quadrupole mass spectrometer in multiple reaction monitoring mode as previously described [47]. The mobile phase consisted of 75/25 of methanol/water with formic acid (0.5%) and 5mM ammonium formate (0.1%) as solvent A and 99/1of methanol/water with formic acid (0.5%) and 5mM ammonium formate (0.1%) as solvent B. The column was equilibrated back to the initial conditions in 3min. The flow rate was 0.5mL/min with a column temperature of 60°C. The sample injection volume was 10 μL. The mass spectrometer was operated in the positive electrospray ionization mode with optimal ion source settings determined by synthetic standards with a declustering potential of 46V, entrance potential of 10V, collision energy of 19V, collision cell exit potential of 14V, curtain gas of 30psi, ion spray voltage of 5,500V, ion source gas1/gas2 of 40psi, and temperature of 550°C. for S1P, total S1P (DH-S1P and S1P) is reported. For C1P, total C1P which is the sum of all C1P species (C2-C1P, C12-C1P, C-14 C1P, C16-C1P, DH-16 –C1P, C18-C1P, C18-1-C1P, C20-C1P, C22-C1P, C24-C1P, C24-C1P, C24-1-C1P, C26-C1P and C26-1-C1P) is reported.

### CXCL12 assessment following G-CSF and Plerixafor therapy

6-8-week-old sphingosine kinase 1 and 2 KO mice as well as appropriate age- and sex-matched C57/Bl6 WT control mice were used for these studies. Mice were mobilized by subcutaneous injection of 100 μg/kg human G-CSF (Amgen, Thousand Oaks, CA) daily for 6 days and a single intraperitoneal injection of Plerixafor 5 mg/kg (Sigma, St. Louis, MO) on the sixth day. Six hours after the last G-CSF injection/2 h post Plerixafor injection, peripheral blood (PB) [47] was obtained from the vena cava (with a 25-gauge needle and 1-mL syringe containing CTAD + EDTA). Plasma was obtained using centrifugation of PB samples at 700g for 10 minutes. Bone marrow (BM) supernatant was isolated from flushed tibia and femur in identical volume (500 µl) of ice-cold PBS. Supernatant was isolated following centrifugation at 700g for 10 minutes. CXCL12 ELISA (DY460 kit) was performed in duplicate on plasma and BM supernatant samples using manufacturer protocol (R&D Systems, Minneapolis, MN, USA) [35].

### Short-term homing experiments

Lethally irradiated experimental mice (γ-irradiation at 1000 cGy) 24 h after irradiation were transplanted (by tail vein injection) with 7 × 10^6^ BM cells from B6-GFP mice. At 24 h after transplant, BM cells from the femurs were isolated via Ficoll-Paque and divided, and 30% of the cells were analyzed by FACS. The rest of the cells were plated in serum-free methylcellulose cultures and stimulated to grow CFU-GM colonies with mGM-CSF (25 ng/ml) and mIL-3 (10 ng/ml). After 7 days of incubation (37°C, 95 % humidity, and 5 % CO_2_), the number of colonies was scored under an inverted microscope [16, 44, 45].

### Evaluation of engraftment

For short-term engraftment experiments, experimental mice were irradiated with 1000 cGy of γ-irradiation. After 24 h, mice were transplanted by tail vein injection with 2.5 × 10^5^ BM cells from WT mice, and the femora of the transplanted mice were flushed with PBS on day 12 post-transplant. After purification via Ficoll-Paque, BM cells were plated in serum-free methylcellulose cultures and stimulated to grow CFU-GM colonies with mGM-CSF (25 ng/ml) and IL-3 (10 ng/ml). After 7 days of incubation (37°C, 95 % humidity, and 5 % CO_2_) the number of colonies was scored under an inverted microscope. Spleens were also removed, fixed in Telesyniczky’s solution for CFU-S assays, and the colonies on the surface of the spleen counted [16, 44, 45].

### Recovery of leukocytes and platelets

Experimental mice were lethally irradiated, and after 24 h the animals were transplanted by tail vein injection with 2 × 10^5^ BM cells from WT mice. Transplanted mice were bled at various intervals from the retro-orbital plexus to obtain samples for white blood cell and platelet counts. Fifty microliters of PB was taken from the retro-orbital plexus of the mice into EDTA-coated Microvette tubes (Sarstedt Inc., Newton, NC, USA) and run within 2 h of collection on a HemaVet 950FS hematology analyzer (Drew Scientific Inc., Oxford, CT, USA) [16, 44, 45].

### Statistical analysis

All results are presented as mean ± SD. Statistical analysis of the data was done using Student’s *t*-test for unpaired samples (Excel, Microsoft Corp., Redmond,WA, USA) with a value of p ≤ 0.05 considered significant.

## SUPPLEMENTARY MATERIALS FIGURES


