# Correction: Mobilization studies in mice deficient in sphingosine kinase 2 support a crucial role of the plasma level of sphingosine-1-phosphate in the egress of hematopoietic stem progenitor cells

**DOI:** 10.18632/oncotarget.26195

**Published:** 2018-09-25

**Authors:** Mateusz Adamiak, Lakshman Chelvarajan, Kevin R. Lynch, Webster L. Santos, Ahmed Abdel-Latif, Mariusz Z. Ratajczak

**Affiliations:** ^1^ Stem Cell Institute at James Graham Brown Cancer Center, University of Louisville, Louisville, KY, USA; ^2^ Department of Regenerative Medicine, Warsaw Medical University, Warsaw, Poland; ^3^ Division of Cardiovascular Medicine, Gill Heart Institute, University of Kentucky, Lexington, KY, USA; ^4^ Department of Pharmacology University of Virginia, Charlottesville, VA, USA; ^5^ Department of Chemistry, Center for Drug Discovery, Virginia Tech, Blacksburg, VA, USA

**This article has been corrected:** The correct Acknowledgments and Funding is provided below:

**ACKNOWLEDGMENTS AND FUNDING**

This work was supported by NIH grants 2R01 DK074720-10 and R01HL112788, the Stella and Henry Endowment, and the Harmonia NCN grant UMO-2014/14/M/NZ3/00475 to MZR, the Preludium NCN grant UMO-2016/23/N/NZ4/03345 to MA, R01GM121075 to KRL and WLS and the UK COBRE Early Career Program (P20 GM103527) to AAL. MA supported by the Foundation for Polish Science (FNP).

Original article: Oncotarget. 2017; 8:65588-65600. https://doi.org/10.18632/oncotarget.19514

