# Supplementary material for: Mobilization studies in mice deficient in sphingosine kinase 2 support a crucial role of the plasma level of sphingosine-1-phosphate in the egress of hematopoietic stem progenitor cells
Source: Oncotarget. 2017 Jul 24;8(39):65588–600. doi: 10.18632/oncotarget.19514 (PMC5630355; doi:10.18632/oncotarget.19514)
Supplement: Supplementary file 1 [file oncotarget-08-65588-s001.pdf]

Mobilization studies in mice deficient in sphingosine kinase 2 support a crucial role of the plasma level of sphingosine-1-phosphate in the egress of hematopoietic stem progenitor cells

SUPPLEMENTARY MATERIALS

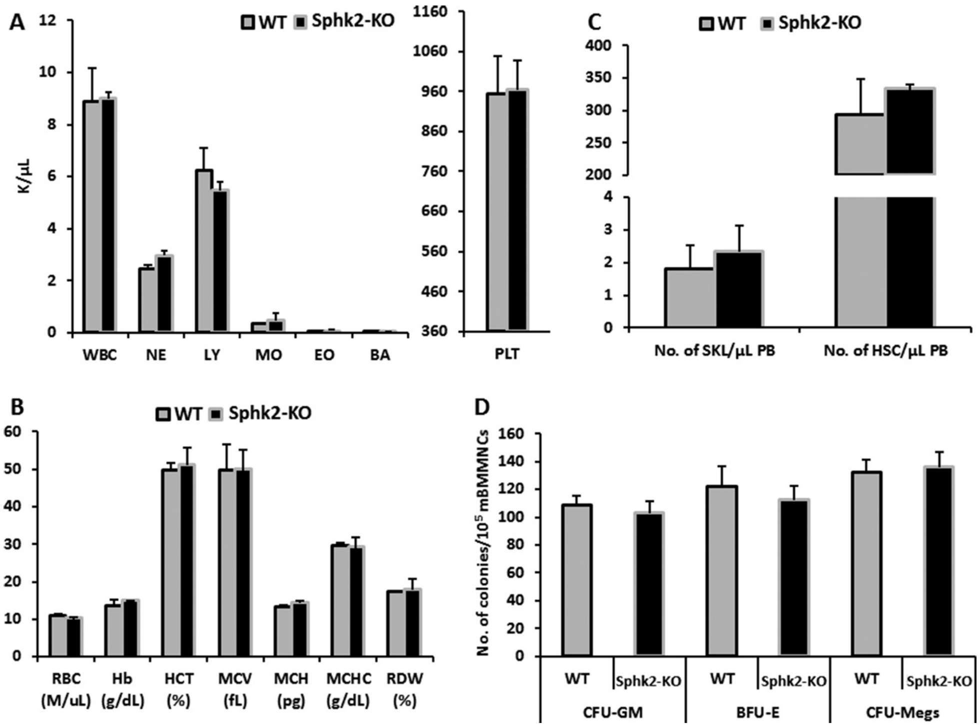

Supplementary Figure 1: Hematological parameters in Sphk2-KO mice. Panel (**A**) PB parameters were evaluated using a HemaVet 950FS analyzer, and Sphk2-KO mice had normal numbers of white blood cells (WBC), neutrophils (NE), lymphocytes (LY), monocytes (MO), and platelets. Panel (**B**) WT mice and Sphk2-KO mice had normal numbers of red blood cells (RBC), hemoglobin content (HB), hematocrit (HCT), mean volume of erythrocytes (MCV), mean content of hemoglobin (MCH), mean concentration of hemoglobin in erythrocytes (MCHC), red cell distribution width (RDW), and platelet counts. Panel (**C**) Under steady-state conditions, there are no differences in the numbers of SKL cells and hematopoietic stem cells (HSCs) circulating in PB between SphK2-KO and WT mice. Panel (**D**) The bone marrow of WT and Sphk2-KO mice was also isolated and evaluated for the numbers of CFU-GM, BFU-E, and CFU-Meg clonogenic progenitors in *in vitro* assays. There were no significant differences in the numbers of clonogenic progenitors in BM between control and Sphk2-KO mice. The data represent an average of at least eight mice tested per experimental group. $*p \leq 0.05$.

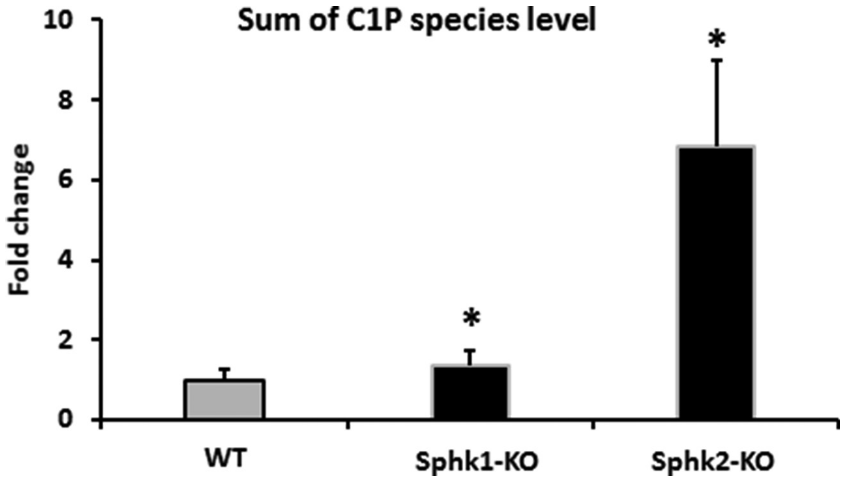

Supplementary Figure 2: Steady-state total C1P plasma levels in Sphk1-KO and Sphk2-KO mice. Steady-state C1P plasma level in WT, Sphk1-KO, and Sphk2-KO mice showing higher levels of C1P in the plasma of Sphk2-KO mice ($N = 4$ animals per group). $*p < 0.005$.
